# A novel oncolytic peptide synergizing with immunotherapy

**DOI:** 10.1080/2162402X.2026.2740845

**Published:** 2026-10-01

**Authors:** Guido Kroemer, Xiaolian Deng, Baldur Sveinbjørnsson, Oliver Kepp

**Affiliations:** a Inserm, Centre de Recherche des Cordeliers, Equipe labellisée Par la Ligue Contre le Cancer, Université Paris Cité, Sorbonne Université, Institut Universitaire de France, Paris, France; b INSERM U23, Université Paris-Saclay, AMMICa, Gustave Roussy, Villejuif, France; c Institut du Cancer Paris CARPEM, Department of Biology, Hôpital Européen Georges Pompidou, AP-HP, Paris, France; d Lytix Biopharma, Oslo, Norway

**Keywords:** Immunotherapy, PD-L1, HMGB1, autophagy

## Abstract

In a recent paper published in Nature, Yuan et al. described an acid-responsive membranolytic peptide that temporally separates lysosomes from later plasma membrane rupture, thereby converting oncolysis into immunogenic cell death. This elegant design permits systemic administration, elicits antigen-specific immunity, and sensitizes tumors to programmed death ligand 1 blockade with remarkably limited toxicity.

Oncolytic peptides are short, usually cationic, amphipathic molecules that preferentially damage malignant cell membranes. Their attraction resides in a double effect: immediate tumor destruction and simultaneous liberation of antigens and danger signals that recruit dendritic cells, prime T lymphocytes, and transform a lesion into an *in situ* vaccine.^1^ Yet brute lysis is not synonymous with immunogenic cell death (ICD). The decisive variable may be the choreography of membrane damage.

Yuan et al.^2^ exploit this distinction using unusual chemical intelligence. The lead, aMPC16-CA50, is derived from a highly helical cationic membranolytic peptide whose side-chain amines are half-masked with citraconic anhydride. At physiological pH, electrostatic repulsion converts the molecule into negatively charged, poorly lytic nanoparticles, limiting the indiscriminate membrane attack. Mild extracellular acidity neutralizes particles and facilitates tumor uptake. Only the more acidic lysosomal lumen cleaves maleic acid amides, restoring positive charge and *α*-helicity. Thus, the charge, conformation, supramolecular assembly, and acid-labile chemistry are not decorative refinements; they constitute a hierarchical safety-and-activation circuit. By varying the anhydride and masking fraction, the investigators tunably shifted the pH threshold and lytic kinetics.^2^ This structure–function matrix is exemplary rational pharmacology.

The conceptual masterstroke is temporal. The activated peptide first ruptures lysosomes, and only approximately 9.5 hours later permeabilizes the plasma membrane. This interval allows the lysosomal content and cellular stress to initiate inflammatory transcription before terminal dissolution. Anionic phospholipids appear to transiently buffer outward-diffusing peptides, thereby retarding plasma membrane rupture. Variants causing immediate lysis were cytotoxic but poorly immunogenic, whereas variants preserving lysosomal rupture followed by delayed surface rupture cross-primed T cells and cooperated with programmed death-ligand 1 (PD-L1) blockade.^2^ Hence, maximal cytotoxicity is not the therapeutic optimum: a dying cancer cell needs sufficient time to become informative.

Does aMPC16-CA50 induce bona fide ICD? Functionally, the answer to this question is compelling. Treated cells released HMGB1, activated autophagic flux and inflammatory programs, enabled dendritic-cell cross-presentation of ovalbumin, and stimulated antigen-specific murine and human T cells. Most importantly, vaccination with peptide-killed syngeneic tumor cells protected immunocompetent mice against subsequent challenge,^2^ the gold-standard assay for ICD.^3^ Moreover, homologous, but not heterologous, distal tumors responded, and therapeutic efficacy diminished after CD4^+^ or CD8^+^ T-cell depletion. These observations transcend the mere inventory of damage-associated molecular patterns.

However, the molecular inventory remains incomplete. Surface calreticulin exposure, extracellular ATP secretion, and eIF2α phosphorylation have not been directly documented. Tumor cell-derived type I interferon secretion was not rigorously demonstrated, although interferon-related transcriptional signatures emerged. The necessity of HMGB1, autophagy, or defined dendritic cell subsets has not been established genetically or pharmacologically. Markers of necroptosis, pyroptosis, and ferroptosis appeared, yet deletion or inhibition of individual execution modules failed to prevent cell death. Calling this process “membranolytic cell death” is operationally useful,^2^ but proclaiming a new regulated death species may be premature. This study proved that an engineered immunogenic trajectory is more convincing than a distinct endogenous death program.

This trajectory explains the interaction with PD-L1 blockade.^2^ Local peptide treatment plus systemic anti-PD-L1 antibodies controlled injected and contralateral tumors, sometimes yielding complete distal regressions at peptide doses tenfold below the locally active dose. The combination increased interferon-gamma-producing CD4^+^ and CD8^+^ lymphocytes, cytotoxic mediators, antigen-processing signatures, and broad activation of the dendritic, natural killer, and myeloid compartments. Mechanistically, the peptide supplies antigenic cargo, inflammatory adjuvanticity, and cross-priming, whereas checkpoint blockade prevents newly mobilized T cells from being neutralized by adaptive immune resistance.^2^ Yet the bridge remains partly schematic: PD-L1 induction, cDC1 dependence, T-cell clonotypes, priming-site requirements, and durable memory after therapeutic cure await investigation. These experiments could convert synergy from empirical observations into a predictive mechanism.

Another major advancement is tolerability. Intravenous aMPC16-CA50 had a maximum tolerated dose above 20 mg/kg, compared to 2.5 mg/kg for unmasked MPC16, and caused no overt biochemical or histological liver or kidney injury at active doses. Systemic treatment cooperated with anti-PD-L1 in subcutaneous, orthotopic, and peritoneal tumor models.^2^ This is remarkable as a membranolytic agent and distinguishes the construct from peptides confined to accessible lesions. However, “well tolerated” in small murine cohorts must not become “safe”: hepatic and renal accumulation is measurable, while chronic toxicology, hemolysis, complement activation, coagulation, cytokine release, and immunogenicity remain to be examined. Equally important, tumor selectivity was inferred from pH-dependent activation and comparative tolerability rather than proven across matched normal tissues. A key next step will therefore be to assess peptide activation and toxicity side-by-side in matched malignant and nonmalignant organotypic human models before clinical translation.

This precedent makes clinical translation plausible (Table 1). DTT-205 and DTT-304 provoke lysosome-centered ICD and tumor-specific memory after local administration,^4^ whereas melittin illustrates both the potency and systemic toxicity of unrestrained membrane lysis.^5^ LTX-401 targets the Golgi apparatus in the first place to induce all hallmarks of ICD.^6^ Most importantly, ruxotemitide (LTX-315; VP-315) perturbs plasma and mitochondrial membranes, releases ATP and HMGB1,^7^ ^,^ ^8^ and has undergone intratumoral phase I characterization in advanced solid tumors, with increased tumor-infiltrating T-cell clonality.^9^ It has also reached phase II evaluation in basal-cell carcinoma and, combined with pembrolizumab, in PD-1/PD-L1-refractory melanoma (NCT04796194); experimental data likewise support cooperation with immune checkpoint inhibition.^10^

**Table 1.** Selected oncolytic peptides.

Name of oncolytic peptide	Mode of action	Systemic or local administration	Preferential cancer targets	Stage of development
aMPC16-CA50	Acid-gated recovery of cationic helicity; lysosomal rupture followed by delayed plasma-membrane rupture and ICD	Systemic intravenous and local intratumoral	Broad solid-tumor models: colorectal (MC38), breast (EMT6, EO771), pancreatic (Panc02)	Preclinical
DTT-205	Lysosomotropic, ROS- and BAX/BAK-associated, largely caspase-independent lytic ICD	Local intratumoral	Colorectal cancer, fibrosarcoma and lung carcinoma models	Preclinical
DTT-304	Lysosomotropic lysis with partial RIPK3–MLKL contribution, ROS generation and ICD	Local intratumoral	Colorectal cancer, fibrosarcoma and lung carcinoma models	Preclinical
LTX-401	Attains and disrupts the Golgi apparatus to later cause partially BAX/BAK-dependent mitochondrial permeabilization to induce ICD	Local intratumoral	Broad effects on fibrosarcoma, hepatocellular carcinoma and lung cancer	Preclinical
Melittin	Amphipathic pore formation and disruption of plasma and mitochondrial membranes; narrow systemic therapeutic window	Local or formulation-dependent systemic delivery (preclinical)	Broad experimental activity, notably triple-negative and HER2-enriched breast cancer	Preclinical
Ruxotemitide (LTX-315; VP-315)	Cationic nine-mer disrupting plasma and mitochondrial membranes, releasing DAMPs and remodeling the tumor microenvironment	Local intratumoral	Accessible solid tumors; basal-cell carcinoma and melanoma	Clinical: phase I and phase II

Abbreviations: DAMPs, danger-associated molecular patterns; ICD, immunogenic cell death; ROS, reactive oxygen species.

The study by Yuan et al., therefore, advances oncolytic peptides from empirically destructive agents to programmable immune medicines. The most important lesson is not that membranes can be ruptured, which has long been evident, but that subcellular location, physicochemical state, and time determine whether the rupture remains sterile or becomes vaccinogenic. If a favorable therapeutic window survives formal pharmacology and larger-animal toxicology, this acid-gated peptide could reconcile two goals often considered antagonistic: systemic reach and controlled oncolysis. The next task is to identify biomarkers of productive lysosomal engagement and to test whether such programming can re-sensitize genuine checkpoint-refractory human cancers.

## Data Availability

This paper does not contain any original data.
